# Association between sleep duration during pregnancy and gestational diabetes mellitus: a systematic review and meta-analysis

**DOI:** 10.3389/fmed.2024.1337492

**Published:** 2024-04-26

**Authors:** Yuandong Li, Chao Liang, Cui Wu, Zheng Nan

**Affiliations:** ^1^Changchun University of Chinese Medicine, Changchun, China; ^2^Affiliated Hospital of Changchun University of Chinese Medicine, Changchun, China

**Keywords:** gestational diabetes, pregnancy, sleep duration, meta-analysis, systematic review

## Abstract

**Objective:**

To systematically review studies on the correlation between sleep duration during pregnancy and gestational diabetes mellitus (GDM) and use meta-analysis to explore the correlation between the two to provide a basis for preventing GDM during pregnancy.

**Methods:**

The search databases were China Knowledge Network (CNKI), Weipu, Wanfang, China Biomedical Literature Service System (SinoMed), Cochrane Library, Web of Science, Embase, and PubMed, and the search time was from the establishment of the above databases to July 2023. The data were statistically analyzed using STATA/MP17 and RevMan 5.3 software. Publication bias could be accurately assessed using funnel plots and Egger’s test.

**Results:**

A total of 5,197 papers were searched, and 13 studies were finally included, which included 80,259 individuals, including 3,461 patients with GDM. The comprehensive analysis showed that. Based on pooled data from prospective, cross-sectional, and case–control studies, extreme sleep duration during pregnancy was strongly associated with GDM compared with average sleep duration. The results of the prospective studies showed that both short (OR = 1.50, 95% CI: 1.07–2.10, *I*^2^ = 60.9%, *p* = 0.02) and long (OR = 1.28, 95% CI: 1.13–1.46, *I*^2^ = 0.0%, *p* < 0.0001) sleep duration increased the risk of gestational diabetes mellitus, but the harms were more pronounced with short sleep. In analyzing the association between extreme sleep duration and GDM, publication bias was found in prospective, cross-sectional, and case–control studies with moderate heterogeneity and prospective-only studies with low heterogeneity.

**Conclusion:**

Both too short and too long sleep duration during pregnancy are strongly associated with GDM. Either too short or too long sleep duration predicts the risk of developing GDM, but the harms are more pronounced with short sleep. These findings remind us of the importance of controlling sleep duration during pregnancy and help to optimize early strategies to prevent GDM.

**Systematic review registration**: http://www.crd.york.ac.uk/prospero, identifier [CRD42023470925].

## 1 Introduction

Pregnant women who had normal glucose metabolism or maybe decreased glucose tolerance before becoming pregnant but developed or were diagnosed with diabetes mellitus during pregnancy are said to have gestational diabetes mellitus (GDM). The prevalence of this disease is reported to be 5.40–33.60% in China (1) and 6.07–20.40% worldwide (2, 3). Numerous factors, including advanced maternal age, obesity, and a family history of diabetes mellitus, increase the chance of developing GDM (4, 5).

Sleep is a necessary physiological need for humans, and a person needs to spend one-third of their life sleeping (6). Adequate and sound sleep during pregnancy is one of the conditions for the mother’s health and the development of the fetus. The National Sleep Foundation’s recommended sleep time for adults is 7–9 h (7). However, it is typical for expectant mothers to feel sleep disturbances during pregnancy because of anxiety, physical discomfort, and hormonal shifts related to childbirth (8). Previous studies have shown that both too much and too little sleep can make type 2 diabetes more likely, and the association between sleep duration and type 2 diabetes has a U-shaped curve (9). A growing number of investigations conducted in recent years have revealed that the occurrence of GDM may be related to sleep duration (10–12), which has aroused extensive attention to the correlation between sleep and GDM in various circles.

There is a relative lack of studies and inconsistent findings on the relationship between sleep duration and GDM in the specific group of pregnant women. A meta-analysis (13) suggested that prolonged sleep may be a pregnant woman’s risk factor for GDM, showing that excessive sleep increased the risk of developing GDM by 28% (OR = 1.28, 95% CI: 1.10–1.49). In contrast, short sleep showed no statistical significance. However, another meta-study (14) showed that pregnant women who slept for short periods (< 6–7 h/night) had a more significant chance of developing GDM than people who had enough sleep, with a 70% increased risk of developing the disease (OR = 1.70, 95% CI: 1.24–2.33). Therefore, the relationship between sleep duration and GDM must be further confirmed.

## 2 Materials and methods

Ethical approval was not required as this study involved a systematic review and meta-analysis of previously published studies. We adhered to the PRISMA guidelines to ensure transparent and comprehensive reporting of our methods and results (15). This paper is registered at PROSPERO with the number CRD42023470925.

### 2.1 Search strategy

To investigate the relationship between sleep duration and GDM risk. A computerized search of China Knowledge Network (CNKI), Weipu, Wanfang, China Biomedical Literature Service System (SinoMed), Cochrane Library, Web of Science, Embase, and PubMed, with the years of search from the establishment of the above databases to July 2023, and using the search strategy of subject words plus free words, with the English search terms as follows: (1) “Diabetes, Gestational,” “Diabetes, Pregnancy-Induced,” “Diabetes, Pregnancy Induced,” “Pregnancy-Induced Diabetes,” “Gestational Diabetes,” “Diabetes Mellitus, Gestational,” “Gestational Diabetes Mellitus”; (2) “Sleep,” “Insomnia,” “Sleep Disorder,” “Sleep Initiation and Maintenance Disorders,” “Sleep Hygiene,” “Dyssomnias,” “Lifestyle,” “Healthy Lifestyle,” “sleep duration.” The search formula can be found in Table 1. Additionally, we screened the reference lists of relevant articles and systematic reviews to identify any additional studies that met our inclusion criteria.

**Table 1:** Specific search strategies in PubMed.

Search	Query
#1	Diabetes, Gestational [Mesh]
#2	Diabetes, Pregnancy-Induced[Title/Abstract] OR Diabetes, Pregnancy Induced[Title/Abstract] OR Pregnancy-Induced Diabetes[Title/Abstract] OR Gestational Diabetes[Title/Abstract] OR Diabetes Mellitus, Gestational[Title/Abstract] OR Gestational Diabetes Mellitus[Title/Abstract]
#3	#1 OR #2
#4	Sleep [Mesh]
#5	Insomnia[Title/Abstract] OR Sleep Disorder[Title/Abstract] OR Sleep Initiation and Maintenance Disorders[Title/Abstract] OR Sleep Hygiene[Title/Abstract] OR Dyssomnias[Title/Abstract] OR Lifestyle[Title/Abstract] OR Healthy Lifestyle[Title/Abstract] OR sleep duration[Title/Abstract]
#6	#4 OR #5
#7	#3 AND #6

### 2.2 Inclusion and exclusion criteria

*Inclusion criteria:* Studies were included in this Meta-analysis if they met the following criteria: (1) participants were pregnant women with undiagnosed diabetes mellitus before pregnancy; (2) observational or experimental studies investigating the relationship between gestational sleep duration and GDM in pregnant women; (3) clinically diagnosed GDM was used as an outcome; (4) The study was designed as a cohort study, a cross-sectional study, and a case–control study, with GDM risk as the primary observation indicator, and was expressed as an adjusted odds ratio (OR); (5) to report the duration of sleep during pregnancy; (6) Published articles in Chinese and English.

**Figure 1:**
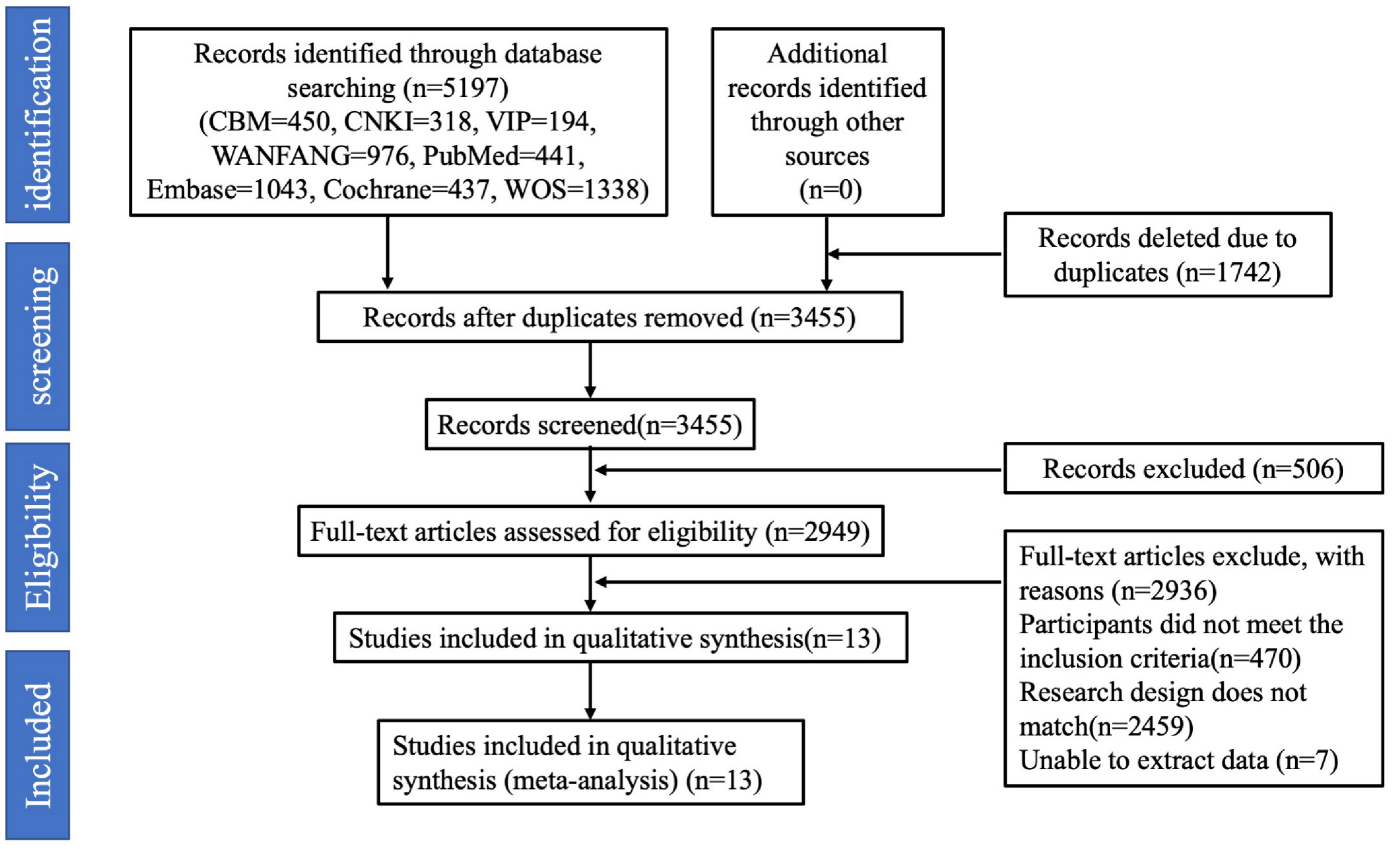
Flow chart of identification of eligible studies.

*Exclusion criteria:* (1) the number of study participants <10; (2) secondary research literature; (3) reviews, case reports, commentaries, etc.; (4) ambiguous reporting of sleep duration, which could not be grouped according to sleep duration; (5) duplicate reporting of the literature; and (6) studies with endpoints other than GDM. The detailed process of identifying eligible studies and the reasons for exclusion are shown in Figure 1.

### 2.3 Data extraction and quality assessment

Two researchers independently extracted data according to the inclusion and exclusion criteria. After reading the complete text, the following information was extracted from each study: (1) first author; (2) year of publication; (3) type of study; (4) country (study site); (5) sample size and number of GDM cases; (6) age; (7) sleep duration measures; (8) clinical diagnostic tools for GDM; and (9) covariates used for adjusted covariates (Table 2). If the adjusted OR had different adjustment levels, the level with the most considerable adjustment was selected. If disagreement was encountered, the agreement was reached in consultation with a third researcher to finalize inclusion in the study.

**Table 2:** Basic characteristics of the included studies.

Study	Design	Country	Sample size	Age, years	GDM, case	Sleep duration		GDM diagnosis		Adjusted confounding factor
						Classification	Period, gestation weeks	Diagnosis criteria	Period, gestation weeks	
Cai et al. (16)	Cross-sectional	Singapore	686	30.7 ± 5.1	131	<6 h ≥6 h	26–28	OGTT	24–28	Maternal age, ethnicity, education, BMI, at <14 weeks of gestation, previous history of gestational diabetes, State–Trait Anxiety Inventory total score
Facco et al. (17)	Prospective cohort	USA	182	29.7 ± 5.5	10	<7 h ≥7 h	13.8 ± 3.8	OGTT, prenatal record	24–28	Age, race/ethnicity, pre-pregnancy BMI, frequent snoring
Facco et al. (18)	Prospective cohort	USA	782	≥18	33	<7 h ≥7 h	15–22	OGTT, medical record	After enrollment and before delivery	BMI
Facco et al. (19)	Prospective cohort	USA	7,668	26.7 ± 5. 6	304	<7 h ≥7 h	6–13, 22–29	OGTT	6–13, 22–29	Age, BMI, race/ethnicity, employed, insurance status
Myoga et al. (20)	Prospective cohort	Japan	48,787	31.2 ± 5.0	1,000	<5 h 5–7 h 7–10 h ≥10 h	16, 27	RBG, GCT, OGTT	The first trimester, the second trimester	Age, pre-pregnancy BMI, weight gain
Qiu et al. (21)	Prospective cohort	USA	1,290	33.3 ± 4. 4	68	≤4 h 5–8 h 9 h ≥10 h	≤20	OGTT, medical record	24–28	Maternal age, race/ethnicity
Rawal et al. (22)	Prospective cohort	USA	2,581	28.1 ± 5.5	107	5–6 h 7 h 8–9 h ≥10 h	8–13, 16–22	OGTT, medical record	After enrollment and before delivery	Maternal age, gestational age, race/ethnicity, parity, education, pre-pregnancy BMI, marital status, family history of diabetes, napping frequency
Reutrakul et al. (23)	Cross-sectional	USA	169	28.5 ± 5.5	26	<7 h ≥7 h	13–27	OGTT	13–27	None
Wang et al. (24)	Prospective cohort	China	12,506	28.5 ± 2.8	919	<7 h 7–9 h ≥9 h	≤12	OGTT	24–28	Maternal age, height, family history of diabetes, parity, Han ethnicity, education, BMI, systolic blood pressure, multiple pregnancies, weight gain, smoking, drinking,
Wang et al. (26)	Case–control	China	500	28.0 ± 3.3	196	<7 h 7–8.9 h 9–9.9 h ≥10 h	1–13	OGTT	24–28	Age, ethnicity, education, drinking, smoking, pre-pregnancy BMI, gestational age, parity, family history of diabetes
Zhong et al. (25)	Prospective cohort	China	4,066	28.3 ± 3.4	335	<7 h 7–8.5 h ≥8.5 h	After enrollment and 24–28 weeks	OGTT	24–28	Pre-gravid BMI, maternal age, family history of diabetes, education, smoking, weight gain
Wang et al. (27)	Case–control	China	500	28.0 ± 3.3	196	< 7 h 7–8.9 h 9–9.9 h ≥10 h	1–13	OGTT	24–28	Age, ethnicity, education, family history of diabetes, drinking, smoking, pre-pregnancy BMI,
Zhou et al. (28)	Prospective cohort	China	542	29.5 ± 4.0	136	<7 h 7–9 h ≥9 h	The gestational age for the first prenatal check-up <12 weeks	OGTT	24–28	Age, pre-pregnancy BMI, weight gain, education, pregnancy time, childbirth, type 2 diabetes family history, smoking, exercise, employment

All studies were evaluated for literature quality by two researchers using the Newcastle-Ottawa scale, which consists of 3 dimensions (selection of study population, comparability, and evaluation of exposure or outcome) (29). Studies with a score of 0–3 are of low quality, 4–6 are moderate quality, and 7–9 are high quality. Studies with scores greater than or equal to 6 were included for analysis.

### 2.4 Statistical analysis

Data were analyzed using RevMan 5.3 and Stata/MP17 software. To determine the association between the risk of extreme sleep duration and GDM, corrected OR and 95% CI information was collected for each trial. The heterogeneity of the studies was analyzed using the *I*^2^ test and Q-test; when *p* < 0.1, heterogeneity was indicated. The presence of low, medium, and high heterogeneity was defined when *I*^2^ reached 25, 50, and 75%, respectively. When *I*^2^ < 50%, the heterogeneity among the literature was small, and the effect sizes were combined using a fixed-effects model. When *I*^2^ > 50%, a random effects model was used, and further subgroup analysis and sensitivity analysis were used to analyze the sensitivity of the included studies. Publication bias could be accurately assessed using funnel plots and Egger’s test. If publication bias existed, the cut-and-patch method was used to determine the effect of publication bias on the outcomes by estimating the actual central value and providing the estimates of the missing studies.

## 3 Results

### 3.1 Search results

A total of 5,197 articles were searched. Firstly, 1,742 duplicates were excluded, 506 irrelevant articles were excluded by reading the titles and abstracts, and then 2,936 articles were excluded by reading the full text of the articles, including reviews, data descriptions, commentaries, case studies, and repetitive publications. Finally, 13 studies were included, 11 in English and 2 in Chinese, and the screening process is shown in Figure 1.

### 3.2 Study characteristics

Literature was published from 2010 to 2021, six from the United States (17–19, 21–23), one from Singapore (16), one from Japan (20), and five from China (24–28). In the current meta-analysis, we evaluated the connection between extreme sleep and the risk of GDM. Based on each preliminary study, we categorized sleep duration into three main categories: short, reference, and long sleep (30). When determining the correlation between extreme sleep duration and the risk of GDM, we combined short and long sleep duration from different studies. The outcome of interest is incident GDM. Assess sleep duration as an exposure factor, objective measurements were recorded using an Actiwatch Spectrum instrument in one paper (18), and self-assessment was used in 12 other articles (16, 17, 19–28). For the assessment of GDM using the OGTT method, Cai et al. (16) and Myoga et al. (20) adopted the 1999 World Health Organization WHO criteria for diagnosing GDM (fasting glucose of 7.0 mmol/L or 2-h OGTT blood glucose of 7.8 mmol/L), and Facco et al. (17) and Zhong et al. (25) were used the criteria of the National Diabetes Data Group. Facco et al. (18, 19) use the following criteria: (1) 100 g oral glucose tolerance test on fasting: fasting blood glucose ≥95 mg/dL, 1 h blood glucose ≥180 mg/dL, 2 h blood glucose ≥155 mg/dL, or 3 h blood glucose ≥140 mg/dL (fulfilling any 2 items); (2) 75 g oral glucose tolerance test on fasting: fasting blood glucose ≥92 mg/dL, 1 h blood glucose ≥180 mg/dL, or 2 h blood glucose ≥153 mg/dL (fulfilling any 1 item); (3) 50 g oral glucose tolerance test on non-fasting: 1 h blood glucose ≥200 mg/dL. Qiu et al. (21) adopted the American Diabetes Association’s (ADF) glucose test. Reutrakul et al. (23) used the criterion of a 50 g glucose tolerance test with a 1 h glucose of ≥200 mg/dL, and three studies (24, 26, 27) used the International Organization for the Study of Diabetes and Pregnancy definition. Blood glucose ≥10.0 mmol/L or 2 h blood glucose ≥8.5 mmol/L. Zhou et al. (28) used the GDM diagnostic criteria in the Chinese Guidelines for the Diagnosis and Treatment of Diabetes Mellitus in Pregnancy and Complicated Gestation (2014) (31) to diagnose GDM. Rawal et al. (22) based their diagnosis of GDM either on the OGTT, using the Carpenter and Coustan diagnostic criteria, or on the medication indications for GDM in hospital fee-for-service diagnosis. All these methods of diagnosing GDM were approved for inclusion in our meta-analysis.

A total sample size of 80,259 patients was included in this study, with 3,461 cases (4.31%) of morbidity. All patients were pregnant, and those with pre-pregnancy diabetes were excluded. Adjustments for confounders varied between studies, but all adjusted for recognized or potential confounders (e.g., BMI, race, sex, age, etc.). Table 2 displays the primary attributes of the listed studies.

### 3.3 Quality assessment

Under the NOS criterion, according to NOS, four studies were of high quality (18, 19, 21, 22), and nine studies were of moderate quality (16, 17, 20, 23–28). All studies provided usable OR and 95% CI.

### 3.4 Extreme sleep duration and GDM

**Figure 2:**
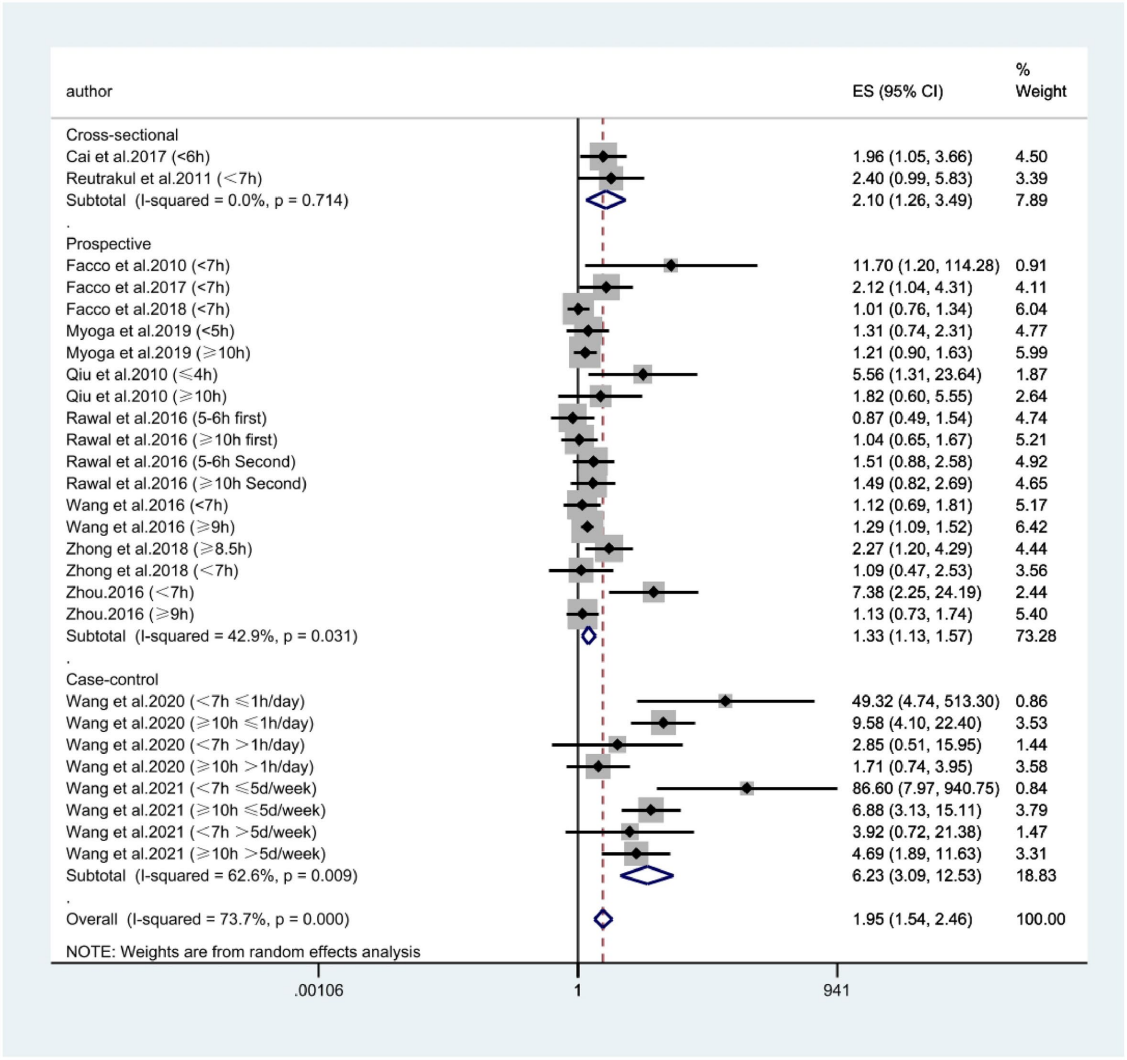
Forest plot of association between extreme sleep duration and GDM, based on pooled data from prospective, cross-sectional, and case–control studies. The results are expressed as odds ratios (OR) and 95% confidence intervals (95% CI). GDM, gestational diabetes mellitus.

**Figure 3:**
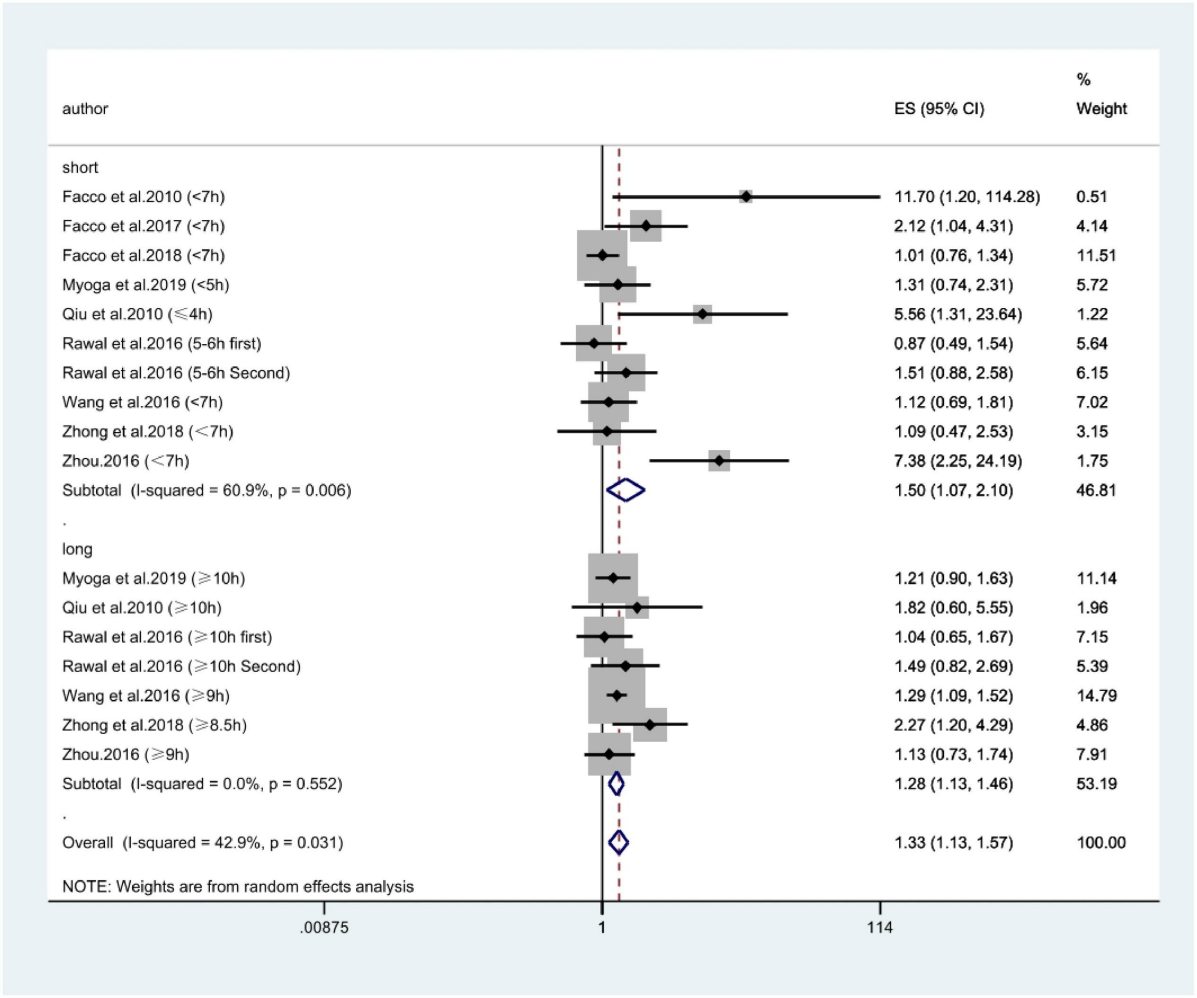
Forest plot of association between extreme sleep duration and GDM, based only on data from prospective studies. The results are expressed as odds ratios (OR) and 95% confidence intervals (95% CI). GDM, gestational diabetes mellitus.

According to Figure 2, combining prospective, cross-sectional and case–control studies, there was a significant relationship between extreme sleep duration during pregnancy and the risk of GDM (combined: OR = 1.95, 95% CI: 1.54–2.46, *I*^2^ = 73.7%, *p* < 0.00001; prospective: OR = 1.33, 95% CI: 1.13–1.57, *I*^2^ = 42.9%, *p* = 0.0006; cross-sectional: OR = 2.10, 95% CI: 1.26–3.49, *I*^2^ = 0.0%, *p* = 0.005; case–control: OR = 6.23, 95% CI: 3.09–12.53, *I*^2^ = 62.6%, *p* < 0.00001); subgroup analyses showed that studies from the United States and Asia demonstrated an association between extreme sleep duration in pregnancy and GDM risk there was an association between (US: OR = 1.44, 95% CI: 1.07–1.92, *I*^2^ = 49%, *p* = 0.01; Asia: OR = 2.37, 95% CI: 1.70–3.32, *I*^2^ = 80%, *p* < 0.00001). Next, we combined nine prospective cohort studies to examine the predictive significance of both short and long sleep duration in the development of GDM. We found that pregnant women with longer sleep duration had an increased risk of GDM (OR = 1.28, 95% CI: 1.13–1.46, *I*^2^ = 0.0%, *p* < 0.0001, Figure 3); the duration of short sleep gestation was also a risk factor for GDM (OR = 1.50, 95% CI: 1.07–2.10, *I*^2^ = 60.9%, *p* = 0.02, Figure 3). This implies that extreme sleep duration during pregnancy may increase the chance of incident GDM.

### 3.5 Publication bias and sensitivity analysis

**Figure 4:**
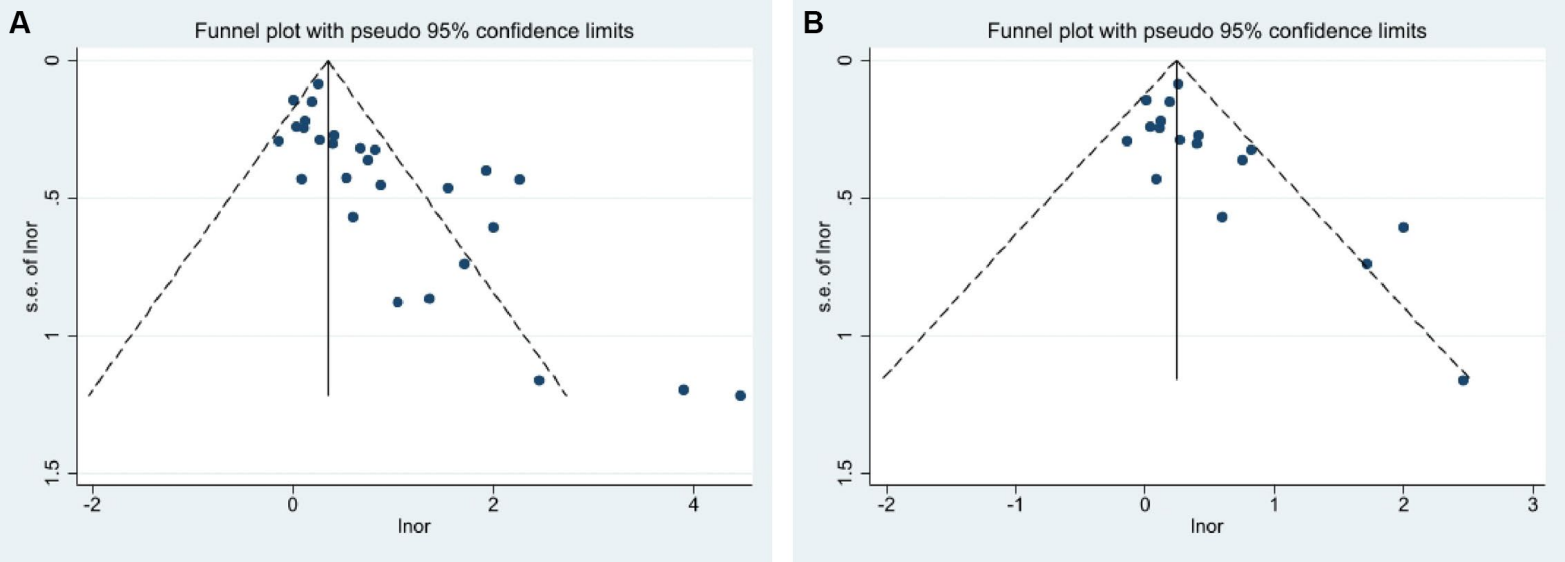
Funnel plots of publication bias analysis **(A)** Combined prospective, cross-sectional, and case–control studies; **(B)** only from prospective studies.

Publication bias was found when combining prospective, cross-sectional, and case–control studies in assessing the predictive role of extreme sleep duration on GDM events (*p* = 0.000, Figure 4A). Combining prospective studies revealed publication bias (*p* = 0.021, Figure 4B). Meta-regression analysis was used to assess the role of covariates on the outcome and discovered that the GDM diagnosis’s definition was not responsible for the heterogeneity (*p* = 0.533). Therefore, the clipping method was used to determine the effect of publication bias on the results by estimating the actual center value and missing studies. There was no publication bias after adding 10 data samples after combining prospective, cross-sectional, and case–control studies through 4 iterations of calculations (the exact number of publications could not be determined because of the variable samples available in each literature). Heterogeneity test: Q = 191.217, *p* = 0.000, using the random effect model, the OR of the combined effect after clipping was 1.34 (95% CI: 1.03–1.75), which did not change significantly, suggesting that the results of the Meta-analysis were robust. Publication bias did not affect the results of the Meta-analysis. Combined with the prospective study, there was no publication bias after adding 4 samples after 4 iterations of calculation and the heterogeneity test: Q = 49.209, *p* = 0.042, using the random effect model, and the OR of the combined effects after clipping and patching was 1.23 (95% CI: 1.01–1.50), which did not change significantly, suggesting that the results of the Meta-analysis were relatively robust. The results of the Meta-analysis were not affected by publication bias. The overall conclusions of the meta-analysis remained unchanged after the literature was excluded one by one using the exclusion method, which suggested that the overall stability of the study was good and the conclusions were reliable.

## 4 Discussion

Sleep, an indispensable physiological need in human life, significantly affects people’s health, especially for some people. Changes in endocrine metabolism in pregnant women during early pregnancy will have both physiological and psychological effects, quickly leading to emotional stress and endocrine hormone imbalance, which in turn affects their sleep conditions (17). Relevant studies have shown that the sleep status of pregnant women is associated with the occurrence of adverse pregnancy outcomes such as preeclampsia and preterm labor (18, 19). GDM is among the most typical pregnancy problems. The occurrence of GDM is influenced by the BMI, age, and family history of diabetes mellitus of pregnant women. The increasing prevalence of GDM has increased the burden of global public health in recent years (32). In this study, we reviewed the studies on the correlation between extreme sleep duration during pregnancy and GDM through a comprehensive search system. We explored the correlation between the two through meta-analysis so that GDM could be prevented better.

Our meta-analysis was based on observing nine cohort studies (17–22, 24, 25, 28), two cross-sectional studies (16, 23), and two case–control studies (26, 27) to provide a link between the risk of GDM and extreme sleep duration during pregnancy. We found a strong association between extreme sleep duration during pregnancy and the risk of GDM, which was confirmed by 80,259 pregnant women and 3,461 patients with GDM and supported by prospective, cross-sectional, and case–control studies from the United States and Asia. Prospective results showed that women who slept too little during pregnancy had a 1.50-fold increased risk of developing GDM. In addition, long sleep duration during pregnancy is also a risk factor for the development of GDM, with a 1.28-fold increased risk of GDM in women with excessive sleep duration during pregnancy. These findings provide a comprehensive overview of the relationship between extreme sleep duration during pregnancy and the risk of GDM and remind us of the importance of controlling sleep duration in pregnant women.

Epidemiologic studies have examined the correlation between sleep during pregnancy and GDM, but the results and conclusions have been inconsistent. The current research found a statistical association between sleep duration and the risk of developing GDM. This result is consistent with some previous studies. Wang et al. (24) found that by analyzing the sleep of 12,506 women in mid-pregnancy in Tianjin, China, the risk of GDM was increased by 21% (95% CI: 1.03–1.42) in pregnant women with sleep duration <7 h compared with those with sleep duration of 7–9 h per day (the sleep duration in this study included daytime sleep duration) The risk of GDM increased by 21% (95% CI: 1.03–1.42) in pregnant women who slept <7 h, and by 61% (95% CI: 1.04–2.50) in those who slept ≥9 h. Other studies have shown a U-shaped correlation between the sleep duration during pregnancy and the risk of GDM, i.e., either too short or too long sleep duration increases the risk of GDM (16, 17). Unlike the results of this study, it has been found that short sleep duration during pregnancy can lead to an increased risk of GDM in pregnant women (23). Compared with pregnant women who slept ≥7 h per night, Facco et al. (18, 19) found an increased risk of GDM in women who slept <7 h per night in both early and mid-pregnancy, with the risk of GDM increasing 1.45-fold (95% CI: 1.10–1.92) and 1.14-fold (95% CI: 1.11–4.53), respectively suggesting that pregnancy may also be an influential factor. In addition, Du et al. (33) found in a cohort study that pregnant women with short night sleep duration (< 7 h) had a 32% increased risk of developing GDM compared to pregnant women in early pregnancy with night sleep duration of 7–9 h. In contrast, those with prolonged sleep duration (> 9 h) showed no statistically significant association. Balserak et al. (34) found that after adjusting for age, ethnicity, and neck circumference, there was no significant correlation between sleep duration during pregnancy and GDM, which is different from the findings of other studies. However, this study observed that either too short or too long sleep duration during pregnancy was associated with an increased risk of GDM onset, but the harm was more pronounced with short sleep. This is inconsistent with two 2018 meta-analyses. Xu et al. (13) concluded that prolonged sleep may be a risk factor for women during pregnancy, and short sleep showed no statistical significance. However, another meta-study by Reutrakul et al. (14) showed that pregnant women with brief duration of sleep (< 6–7 h/night) were more likely to develop GDM than those with adequate sleep duration. In the present study, we found that pregnant women with the shortest limit of sleep duration during pregnancy were less than 4 h and generally within 7 h. The most extended limit of sleep duration was usually more than 10 h, except for Zhong et al. (25), who showed that the most extended sleep duration was ≥8.5 h. We could observe that the optimal sleep duration of pregnant women during pregnancy in the 13 papers included was generally in the range of 7–9 h, like the idea of 8 h of optimal sleep time that we are familiar with daily.

Possible reasons for the differences between this study and previous studies include, first, the existence of racial specificity in the prevalence of GDM (35). Second, there is no standardized definition of the length of sleep duration at night, and different studies have different criteria and reference standards for the division of sleep duration. In addition, in this study, due to the limitation of sample size, there were relatively few pregnant women with extreme sleep duration (too short or too long). The scope of the division of sleep periods was narrower than that of other studies, which may affect the research results and lead to consistency with previous studies. Thirdly, different diagnostic criteria for GDM used in various studies may lead to differences in the results: there are one-step and two-step tests for GDM, and there are IADPSG, American Diabetes Association (ADA), European Association for the Study of Diabetes (EASD), etc. (36). Fourth, due to the limitations of research conditions, achieving objective standardization in measuring sleep duration takes a lot of work. The sample sizes of studies that have been able to use instruments to measure sleep duration at night objectively are generally small, and most of the studies with large sample sizes have used self-reporting by respondents relying on their memories, which may result in bias and influence the results and lead to discrepancies. Therefore, more extensive prospective cohort studies using scientifically standardized measurement instruments must demonstrate the association between sleep duration and GDM.

The strengths of this study: Firstly, the literature included in this study is of relatively high quality, and the conclusions are credible. Secondly, meticulous adjustments for multiple confounding variables within the prior literature integrated into this study enhance the validity of conclusions, ensuring relevance to real-world scenarios. Thirdly, this study diverges from previous research, by investigating the correlation between sleep duration and gestational diabetes mellitus (GDM) during pregnancy, thereby opening avenues for novel perspectives in exploring the impact of sleep duration on GDM.

The limitations of this study: Primarily, despite the substantial overall sample size, the original quantity of studies is comparatively limited, with research sites confined to the United States and Asia. Secondly, inherent publication biases pose challenges when scrutinizing the link between sleep duration during pregnancy and the development of GDM across various study designs. Thirdly, while meta-regression analysis suggests the definition of GDM diagnosis is not solely accountable for heterogeneity, potential covariates, such as interactions between sleep duration, sleep disturbances, or nap times, alongside pre-pregnancy diabetes history and other GDM-related risk factors, remain influential and warrant further investigation. Fourthly, the varying diagnostic criteria for GDM may increase the clinical heterogeneity of this meta-analysis.

The biological mechanisms by which sleep duration affects GDM have yet to be elucidated fully. The mechanisms identified so far include the following: first, excessive or short sleep duration can lead to endothelial dysfunction with increased inflammation and oxidative stress, and endothelial dysfunction is closely related to insulin resistance in type 2 diabetes mellitus (37, 38). Secondly, sleep disorders cause a decrease in basal glucagon concentration, leading to abnormal blood glucose levels (39). Again, Elevated sympathetic nervous system activity, dysregulation of the hypothalamic–pituitary axis (HPA), altered synthesis and release of cytokines and adipokines, high serum cortisol levels, and constriction of peripheral vasculature are other possible biological mechanisms (40, 41).

Although GDM is associated with some risk factors, such as obesity, the exact mechanism remains to be tested. Like type 2 diabetes mellitus, GDM is associated with impaired insulin secretion and insulin resistance, which share the same risk factors and genetic predisposition. Mild insulin resistance is often present in pregnant women due to hormonal changes during pregnancy (42). Therefore, small changes in sleep factors may increase the risk of GDM in pregnant women, and the following mechanisms are hypothesized. Too little sleep may increase the inflammatory response by increasing C-reactive protein concentrations, interleukin-6, and tumor necrosis factor α (43, 44).

Meanwhile, sleep-disordered breathing with intermittent hypoxia during sleep may lead to an increase in oxidative stress, which may lead to hyperactivation of the hypothalamic–pituitary–adrenal axis, which may increase the production of glucocorticoids and cortisol, which are involved in the development and progression of insulin resistance (45). Further research into the potential mechanisms linking sleep duration and GDM would be valuable in promoting public health and guiding healthy lifestyles during pregnancy.

## 5 Conclusion

Extreme sleep duration during pregnancy is strongly associated with the development of GDM. Pregnant women who sleep too short or too long during pregnancy have a higher risk of developing GDM. Long and short sleep duration are risk factors for the development of GDM, but the harm is more pronounced with short sleep. These results extend our understanding of the adverse effects of extreme sleep duration during pregnancy and remind us of the importance of controlling sleep duration during pregnancy to improve maternal and infant health. Future well-designed prospective cohort and experimental studies are needed to confirm the current findings and provide additional evidence for the role of sleep duration in the development of GDM.

## Data availability statement

The original contributions presented in the study are included in the article/supplementary material, further inquiries can be directed to the corresponding author.

## Author contributions

YL: Data curation, Formal analysis, Investigation, Methodology, Writing – original draft. CL: Formal analysis, Funding acquisition, Methodology, Software, Validation, Writing – review & editing. CW: Data curation, Formal analysis, Methodology, Validation, Visualization, Writing – review & editing. ZN: Conceptualization, Project administration, Supervision, Writing – review & editing.
